# Supplementary material for: A UMLS-based spell checker for natural language processing in vaccine safety
Source: BMC Med Inform Decis Mak. 2007 Feb 12;7:3. doi: 10.1186/1472-6947-7-3 (PMC1805499; doi:10.1186/1472-6947-7-3)
Supplement: Additional file 13 — RAP application directory. Sets up directory for RAP (RDF application for PHP) source code [file 1472-6947-7-3-S13.gz › rap/doc/phpdoc/__filesource/fsource_resModel__resModelResList.php.html]

xml version="1.0" encoding="iso-8859-1"?


File Source for ResList.php


# Source for file ResList.php

Documentation is available at ResList.php

```
1. <?PHP
2. // ----------------------------------------------------------------------------------
3. // Class: ResList
4. // ----------------------------------------------------------------------------------


8. /**
9. * Implementation of an rdf:Collection (rdf:List)
10. * Provides a convenience encapsulation for lists formed from
11. * chains of RDF statements arranged to form a head/tail cons-cell
12. * structure.
13. *
14. * A well-formed list has cells that are made up of three statements:
15. * one denoting the rdf:type of the list cell, one denoting the link
16. * to the value of the list at that point, and one pointing to the
17. * list tail. If a list cell is not well-formed, list operations may
18. * fail in unpredictable ways. However, to explicitly check that the
19. * list is well-formed at all times is expensive, but you can call
20. * the isValid() method to manually check, if the list is well formed.
21. *
22. *
23. * <BR><BR>History:<UL>
24. * <LI>10-01-2004                : First version of this class.</LI>
25. *
26. * @version  V0.9.1
27. * @author Daniel Westphal <mail at d-westphal dot de>
28. *
29. * @package resModel
30. * @todo nothing
31. * @access    public
32. */
33. class ResList extends ResResource
34. {
35. /**
36. * Holds a ResResource with the uri rdf:rest
37. * @var        ResResource
38. * @access    private
39. */
40. var $rdfRestResource;

42. /**
43. * Holds a ResResource with the uri rdf:first
44. * @var        ResResource
45. * @access    private
46. */
47. var $rdfFirstResource;

49. /**
50. * Holds a ResResource with the uri rdf:nil
51. * @var        ResResource
52. * @access    private
53. */
54. var $rdfNilResource;


57. /**
58. * Constructor
59. * You can supply a URI
60. *
61. * @param string $uri
62. * @access    public
63. */
64. function ResList($uri = null)
65. {
66. //call the parent's constructor
67. parent::ResResource($uri);
68. //initialize vars
69. $this->rdfRestResource = new ResResource(RDF_NAMESPACE_URI.RDF_REST);
70. $this->rdfFirstResource = new ResResource(RDF_NAMESPACE_URI.RDF_FIRST);
71. $this->rdfNilResource = new ResResource(RDF_NAMESPACE_URI.RDF_NIL);
72. }

74. /**
75. * Returns the value of the list element at the specified position or null.
76. *
77. * @param    integer    $position
78. * @return    ResResource
79. * @access    public
80. */
81. function get($position)
82. {
83. //init
84. $listElement=$this;
85. //walk through the list until the position in the list is reached
86. for ($i=0;$i<$position;$i++)
87. {
88. $listElement=$this->_getRestElement($listElement);
89. if($listElement===null)
90. return null;
91. }
92. //return the associated value
93. return $this->_getValue($listElement);
94. }

96. /**
97. *  Add the given value to the end of the list.
98. *  it is only defined if this is not the empty list.
99. *
100. * @param    object ResResource    $resource
101. * @return    boolean
102. * @access    public
103. */
104. function add($resource)
105. {
106. //return false if this list is the empty list
107. if($this->uri==RDF_NAMESPACE_URI.RDF_NIL)
108. return false;

110. //if this is the first value
111. if ($this->isEmpty())
112. {
113. $newLastElement =& $this;
114. } else
115. //if there are other values in the list
116. {
117. //get the last list element
118. $lastElement=$this->_getListElement();
119. //remove the rdf:rest property
120. $lastElement->removeAll($this->rdfRestResource);
121. //create a new list element
122. $newLastElement=$this->model->createResource();
123. //concatenate the new list element with the list
124. $lastElement->addProperty($this->rdfRestResource,$newLastElement);
125. }
126. //add the value
127. $newLastElement->addProperty($this->rdfFirstResource,$resource);
128. //ad the rdf:nil property to the last list element
129. $newLastElement->addProperty($this->rdfRestResource,$this->rdfNilResource);

131. return true;
132. }

134. /**
135. *  Update the head of the list to have the given value,
136. *  and return the previous value.
137. *
138. * @param    object ResResource    $value
139. * @return    ResResource
140. * @access    public
141. */
142. //todo: error handling, when empty list

144. function setHead($value)
145. {
146. //save the old value
147. $oldValue=$this->getHead();
148. //remove the old value
149. $this->removeAll($this->rdfFirstResource);
150. //add the new value
151. $this->addProperty($this->rdfFirstResource,$value);

153. //return the old value
154. return $oldValue;
155. }

157. /**
158. * Get the value that is associated with the head of the list.
159. *
160. * @return    ResResource
161. * @access    public
162. */
163. //todo: error handling, falls empty list

165. function getHead()
166. {
167. return $this->_getValue($this);
168. }

170. /**
171. *  Remove the head of the list. The tail of the list
172. *  remains in the model. Note that no changes are made to
173. *  list cells that point to this list cell as their tail.
174. *
175. * @return    ResList
176. * @access    public
177. */
178. function removeHead()
179. {
180. //get the second list element
181. $rest=$this->_getRestElement($this);
182. //remove the first element
183. $this->removeAll($this->rdfFirstResource);
184. $this->removeAll($this->rdfRestResource);
185. //change this Resource URI to that of the second list element
186. //thus makin it the fist element
187. $this->uri=$rest->getURI();

189. //return the new list
190. return $this;
191. }

193. /**
194. * Get the Position of the first occurrence of the given value in the list,
195. * or -1 if the value is not in the list.
196. * You can supply an offset to search for values. (First element has offset 0)
197. * Default is 0
198. *
199. * @param    object ResResource    $resource
200. * @param    integer    $offset
201. * @return    integer
202. * @access    public
203. */
204. function indexOf($resource, $offset = 0)
205. {
206. //init
207. $element=$this;
208. $actualIndex=0;

210. //walk through the list until the value is found and the position is higher than
211. //the offset
212. while ($actualIndex < $offset || !$resource->equals($this->_getValue($element)))
213. {
214. //get next list element
215. $element=$this->_getRestElement($element);
216. $actualIndex++;

218. //if the end of the list is reached and the value isn't found
219. if ($element===null)
220. return null;
221. }
222. //return the index value
223. return $actualIndex;
224. }

226. /**
227. * Replace the value at the i'th position in the list with the given value
228. *
229. * @param    integer                $index
230. * @param    object ResResource    $resource
231. * @return    object ResResource
232. * @access    public
233. */
234. function replace($index, $resource)
235. {
236. //get the list element at the $index position
237. $listElement=$this->_getListElement($index);
238. //get the old value
239. $oldValue=$this->_getValue($listElement);
240. //remove the old value
241. $listElement->removeAll($this->rdfFirstResource);
242. //add the new value
243. $listElement->addProperty($this->rdfFirstResource,$resource);
244. //return the old value
245. return $oldValue;
246. }

248. /**
249. * Answer true if the given node appears as the value of a value
250. * of any of the cells of this list.
251. *
252. * @param    object ResResource    $value
253. * @return    boolean
254. * @access    public
255. */
256. function contains($value)
257. {
258. //return true, if a position was found.
259. $result=$this->indexOf($value);
260. return ($result!==null);
261. }

263. /**
264. * Get the list that is the tail of this list.
265. *
266. * @return    object ResList
267. * @access    public
268. */
269. function getTail()
270. {
271. //get the second list element
272. $nextListElement= $this->_getRestElement($this);
273. //return the second element as new list
274. return $this->model->createList($nextListElement->getURI());
275. }

277. /**
278. * Remove all of the components of this list from the model.
279. * Note that this is operation is only removing the list cells
280. * themselves, not the resources referenced by the list -
281. * unless being the object of an rdf:first  statement is the
282. * only mention of that resource in the model.
283. *
284. * @return    boolean
285. * @access    public
286. */
287. function removeList()
288. {
289. $element=$this;

291. while ($element!==null)
292. {
293. $nextElement=$this->_getRestElement($element);

295. $element->removeAll($this->rdfFirstResource);
296. $element->removeAll($this->rdfRestResource);

298. if (($nextElement !== null) && ($nextElement->getURI()!==RDF_NAMESPACE_URI.RDF_NIL))
299. {
300. $element=$nextElement;
301. } else
302. {
303. return true;
304. }
305. }
306. return false;
307. }

309. /**
310. * Returns true, if this list is empty
311. *
312. * @param    object Statement    $statement
313. * @return    integer
314. * @access    public
315. */
316. function isEmpty()
317. {
318. return !$this->hasProperty($this->rdfFirstResource);
319. }

321. /**
322. * Get all values in the list as an array of ResResources
323. *
324. * @return    array
325. * @access    public
326. */
327. function getContentInArray()
328. {
329. $result=array();
330. $element=$this;

332. while ($element!==null)
333. {
334. //add the value of the current element to the result if is set.
335. $value=$this->_getValue($element);
336. if ($value!==null)
337. $result[]=$value;

339. //walk through the list until it's end
340. $nextElement=$this->_getRestElement($element);
341. if (($nextElement !== null) && ($nextElement->getURI()!==RDF_NAMESPACE_URI.RDF_NIL))
342. {
343. $element=$nextElement;
344. } else
345. {
346. break;
347. }
348. }
349. //return the result
350. return $result;
351. }

353. /**
354. * Change the tail of this list to point to the given list, so that this list
355. * becomes the list of the concatenation of the elements of
356. * both lists. This is a side-effecting operation on this list;
357. * for a non side-effecting alternative, see append.
358. *
359. *
360. * @param    object ResList    $ResList
361. * @access    public
362. */
363. function concatenate($ResList)
364. {
365. //get the last list element
366. $lastElement=$this->_getListElement();
367. //remove the old tail (rdf:nil)
368. $lastElement->removeAll($this->rdfRestResource);
369. //add the $ResList as new tail
370. $lastElement->addProperty($this->rdfRestResource,$ResList);
371. }

373. /**
374. * Answer a new list that is formed by adding each element of
375. * this list to the head of the given list. This is a non
376. * side-effecting operation on either this list or the given
377. * list, but generates a copy of this list. For a more storage
378. * efficient alternative, see concatenate
379. *
380. * @param    object ResList    $ResList
381. * @return    object ResList
382. * @access    public
383. */
384. function append($resList)
385. {
386. //get a copy of this list
387. $newList=$this->copy();

389. //add all values from the $resList to the new list
390. foreach ($resList->getContentInArray() as $value)
391. {
392. $newList->add($value);
393. }
394. //return the new list
395. return $newList;
396. }

398. /**
399. * Answer a list that contains all of the elements of this
400. * list in the same order, but is a duplicate copy in the
401. * underlying model.
402. *
403. * @return    object ResList
404. * @access    public
405. */
406. function copy()
407. {
408. //create a new list in the model
409. $newList=$this->model->createList();
410. //add all values from this list to the new list
411. foreach ($this->getContentInArray() as $value)
412. {
413. $newList->add($value);
414. }
415. //return the new list
416. return $newList;
417. }

419. /**
420. * Return a reference to a new list cell whose head is value
421. * and whose tail is this list.
422. *
423. * @param    object ResResource    $value
424. * @return    object ResList
425. * @access    public
426. */
427. function cons($value)
428. {
429. //create a new list
430. $newList=$this->model->createList();
431. //add the new value
432. $newList->add($value);
433. //set this list as the tail of the new list
434. $newList->setTail($this);

436. //return the new list
437. return $newList;
438. }

440. /**
441. * Update the list cell at the front of the list to have the
442. * given list as tail. The old tail is returned, and remains
443. * in the model.
444. *
445. * @param    object ResList    $resList
446. * @return    object Reslist
447. * @access    public
448. */
449. function setTail($resList)
450. {
451. //save the old tail
452. $oldTail=$this->getTail();
453. //remove the old tail
454. $this->removeAll($this->rdfRestResource);
455. //add the $resList as new Tail
456. $this->addProperty($this->rdfRestResource,$resList);

458. //return the old tail
459. return $oldTail;
460. }

462. /**
463. * Answer true if this list has the same elements in the
464. * same order as the given list. Note that the standard equals
465. * test just tests for equality of two given list cells.
466. * While such a test is sufficient for many purposes, this
467. * test provides a broader equality definition, but is
468. * correspondingly more expensive to test.
469. *
470. * @param    object ResList    $resList
471. * @return    boolean
472. * @access    public
473. */
474. function sameListAs($resList)
475. {
476. //init
477. $indexPos=0;
478. do
479. {
480. //get the values for both lists at the actual position
481. $thisValue=$this->get($indexPos);
482. $thatValue=$resList->get($indexPos);
483. //if the values aren't equal, return false
484. if (($thisValue !== null) && !$thisValue->equals($thatValue))
485. return false;

487. $indexPos++;
488. //walk until this list reaches a null value (end)
489. } while ($thisValue!==null);

491. //if the other list has a null value at this position too, return true
492. //else return false
493. return ($thatValue===null);
494. }

496. /**
497. * Remove the given value from this list.
498. * If value does not occur in the list, no action is taken. Since removing the
499. * head of the list will invalidate the list head cell, in
500. * general the list must return the list that results from
501. * this operation. However, in many cases the return value
502. * will be the same as the object that this method is invoked
503. * on.
504. *
505. * @param    object ResResource    $value
506. * @return    object ResList
507. * @access    public
508. */
509. function remove($value)
510. {
511. //if the value is the value of the first list element(head)
512. //call the remove head position and return the new head
513. if ($value->equals($this->_getValue($this)))
514. return $this->removeHead();

516. $element=$this;
517. do
518. {
519. $newElement=$this->_getRestElement($element);

521. //if the value equals the value of the current list element
522. if ($newElement !== null && $value->equals($this->_getValue($newElement)))
523. {
524. //remove the link to the list element to be removed
525. $element->removeAll($this->rdfRestResource);
526. //add a link to the list element AFTER the element to be deleted
527. $element->addProperty($this->rdfRestResource,$this->_getRestElement($newElement));
528. //remove the list element with values
529. $newElement->removeAll($this->rdfFirstResource);
530. $newElement->removeAll($this->rdfRestResource);
531. //return this ResList
532. return $this;
533. }
534. $element=$newElement;
535. } while ($element!==null);
536. //return this list
537. return $this;
538. }

540. /**
541. * Answer true if the list is well-formed, by checking that each
542. * node is correctly typed, and has a head and tail pointer from
543. * the correct vocabulary
544. *
545. * @return    boolean
546. * @access    public
547. */
548. function isValid()
549. {
550. $element=$this;
551. if ($this->_getValue($this)=== null && $this->_getRestElement($this) === null)
552. return true;
553. do
554. {
555. //return true if the last element is a rdf:nil
556. if ($element->getURI() == RDF_NAMESPACE_URI.RDF_NIL)
557. return true;
558. //return false, if the current element has no associated value
559. if ($this->_getValue($element) === null)
560. return false;

562. $element=$this->_getRestElement($element);
563. } while ($element !== null);
564. //return false, if the current element has no rdf:rest property
565. return false;
566. }

568. /**
569. * Get the associated rdf:rest Resource from the suplied ResList element
570. *
571. * @param    object ResList    $listElement
572. * @return    object ResList
573. * @access    private
574. */
575. function _getRestElement($listElement)
576. {
577. //get the rdf:rest property
578. $statement= $this->model->getProperty($listElement,$this->rdfRestResource);
579. //return null, if this property isn't set
580. if ($statement === null)
581. return null;

583. //return the value of the rdf:rest property
584. return $statement->getObject();
585. }

587. /**
588. * Returns the list element at the $index position.
589. *
590. * If to $index is suplied, the last list element will be returned

592. * @param    integer    $index
593. * @return    object ResResource
594. * @access    private
595. */
596. function _getListElement($index = null)
597. {
598. $element=$this;
599. $actualIndex=0;

601. while ($element!=null)
602. {
603. //return the current element if index matches the current index
604. if ($actualIndex === $index)
605. return $element;

607. //return the current element if it the last one
608. if ($element->hasProperty($this->rdfRestResource,$this->rdfNilResource))
609. return $element;

611. $nextElement=$this->_getRestElement($element);

613. if ($nextElement!==null)
614. {
615. $element=$nextElement;
616. $actualIndex++;
617. } else
618. {
619. break;
620. }
621. }
622. return $element;
623. }

625. /**
626. * Get the value associated to the $listResource by the rdf:first property
627. *
628. * @param    object ResList    $listResource
629. * @return    object ResResource
630. * @access    private
631. */
632. function _getValue($listResource)
633. {
634. //Return the value of the rdf:first property or null, if it isn't set
635. $statement=$this->model->getProperty($listResource,$this->rdfFirstResource);
636. if ($statement===null)
637. return null;

639. return $statement->getObject();
640. }
641. }
642. ?>
```

Documentation generated on Thu, 7 Jul 2005 13:43:37 +0200 by phpDocumentor 1.3.0RC3
